# Real-world use of denosumab and bisphosphonates in patients with solid tumours and bone metastases in Germany

**DOI:** 10.1007/s00520-020-05357-5

**Published:** 2020-02-21

**Authors:** Ingo Diel, Sonja Ansorge, David Hohmann, Christina Giannopoulou, Daniela Niepel, Michele Intorcia

**Affiliations:** 1 Centre for Gynecological Oncology, Praxisklinik am Rosengarten, Augustaanlage 7-11, 68165 Mannheim, Germany; 2 Vilua Healthcare GmbH, Munich, Germany; 3 Amgen GmbH, Munich, Germany; 4 Amgen Europe GmbH, Rotkreuz, Switzerland; 5 Amgen (Global), Rotkreuz, Switzerland

**Keywords:** Bisphosphonates, Bone metastases, Compliance, Denosumab, Discontinuation, Persistence

## Abstract

**Purpose:**

Bisphosphonates and denosumab prevent bone complications in patients with bone metastases from solid tumours. This retrospective, longitudinal, cohort study provides data on their real-world use in this setting in Germany.

**Methods:**

Adults with bone metastases from breast, prostate or lung cancer who were newly initiated on a bisphosphonate or denosumab between 1 July 2011 and 31 December 2015 were identified from a German healthcare insurance claims database. Primary outcomes included persistence, compliance, discontinuation and switch rates at 12 months.

**Results:**

This study included 1130 patients with bone metastases: 555 (49%) had breast cancer, 361 (32%) prostate cancer and 242 (21%) lung cancer. Mean age was 65 years for patients with breast or lung cancer and 74 years for those with prostate cancer. Across all tumour types, compared with any bisphosphonate, 12-month persistence was higher with denosumab (breast cancer 78% vs 54–58%, prostate cancer 58% vs 50%, lung cancer 68% vs 34–60%), median time to discontinuation was longer with denosumab and switch rates were lower for denosumab (breast cancer 5% vs 14–19%, prostate cancer 2% vs 11%, lung cancer 3% vs 7–12%). Compliance at 12 months was longer for denosumab than for any bisphosphonate in breast cancer (75% vs 42–48%) and in prostate cancer (47% vs 36%).

**Conclusions:**

Patients initiated on denosumab following a diagnosis of bone metastases from breast, prostate or lung cancer had greater medication persistence, longer time to discontinuation, improved compliance and lower switch rates than those initiated on a bisphosphonate.

**Electronic supplementary material:**

The online version of this article (10.1007/s00520-020-05357-5) contains supplementary material, which is available to authorized users.

## Introduction

Bone metastases are common in patients with advanced solid tumours [1–3]; they affect 68% of patients with prostate cancer (> 90% of patients with metastatic castration-resistant disease), 73% with breast cancer and 36% with lung cancer [2, 3]. Bone metastases can lead to debilitating bone complications, known as skeletal-related events (SREs), which include pathologic fractures, spinal cord compression and the need for radiation or surgery to the bone [4]. Bone complications can cause pain, reduce patient quality of life (QoL) and be associated with an increased risk of death [5–8]. They are also linked to high healthcare resource use, thereby placing a considerable burden on healthcare systems [9]. Early treatment to prevent bone complications is, therefore, paramount; results from real-world studies and randomised controlled trials (RCTs) in breast cancer suggest that, on average, a first bone complication occurs as early as 8–9 months after a diagnosis of bone metastases [10, 11].

As the cancer treatment landscape evolves and survival outcomes improve, the long-term implications of supportive care become increasingly relevant to clinical decision-makers. Growing awareness of the prevalence and impact of bone complications has led to greater emphasis on their prevention within clinical cancer guidelines [5, 12, 13]. Bisphosphonates (e.g. zoledronic acid, pamidronate disodium, ibandronate and clodronate) and denosumab are approved for prevention of bone complications in patients with advanced malignancies involving bone [14–18]. Bisphosphonates are synthetic analogues of pyrophosphonate (a natural regulator of bone metabolism) that are incorporated into the bone, and reduce bone resorption by inhibiting the differentiation and activation of osteoclasts [14, 16–20]. They are administered as an intravenous (IV) infusion every 3–4 weeks (zoledronic acid, pamidronate disodium, ibandronate) or orally every day (ibandronate and clodronate) [14, 16–19]. Denosumab is a fully human, monoclonal antibody against the receptor activator of nuclear factor kappa B (RANK) ligand (RANKL). By disrupting RANK signalling, denosumab prevents the fusion and activation of osteoclasts, thus reducing bone resorption [15, 21]. Denosumab is administered as a subcutaneous (SC) injection every 4 weeks [15]. Bisphosphonates and denosumab have different pharmacokinetic profiles owing to their differing mechanisms of action; zoledronic acid has a half-life of 2–189 days and may remain in bone for up to 10 years, whereas denosumab is not incorporated into the bone and has a mean half-life of 14–55 days [14, 15, 22]. Bisphosphonates and denosumab reduce the incidence of bone complications in patients with bone metastases from solid tumours [23–25], with denosumab showing superiority compared with zoledronic acid in patients with breast [26] and prostate cancer [27] and non-inferiority in patients with multiple myeloma and other solid tumours [28]. These agents have also been shown to prevent pain progression and the worsening of patient QoL [5], with denosumab shown to be more effective than zoledronic acid in these dimensions [29].

The European Society for Medical Oncology recommends that a bisphosphonate or denosumab is initiated at the time of bone metastasis diagnosis to reduce pain and delay bone complications [5]. Clinical guidelines draw heavily on RCTs to provide robust evidence on the safety and efficacy of therapies. RCTs offer high internal validity, but their findings are based on the use of agents in closely managed, highly selected patients often treated over the short term [30]. In contrast, routine clinical practice typically involves diverse patient populations and care settings, and long-term treatment periods. Thus, the use and effectiveness of therapies must also be evaluated in real-world practice. Importantly, the full benefit of medications will only be achieved if patients follow prescribed regimens [31]; understanding treatment persistence and compliance is critical to interpretation of real-world outcomes. Persistence can be defined as the duration of time from initiation to discontinuation of therapy, whereas compliance can be defined as the extent to which a patient acts in accordance with the prescribed interval and dosing of a regimen [32]. Two studies using data from the Oncology Services Comprehensive Electronic Records (OSCER) in the United States of America (USA) have shown that in patients with bone metastases from solid tumours, compliance and persistence with denosumab are higher than with IV bisphosphonates [33, 34]. To build on these findings from a European perspective, this retrospective study was conducted to provide real-world data on bisphosphonate and denosumab usage (persistence, compliance, switching and drug re-initiation rates) in a large sample of patients with bone metastases, from breast, prostate or lung cancer, receiving current anti-tumour treatment in routine clinical practice in Germany. Exploratory analysis of potential implications of time to initiation of denosumab or a bisphosphonate on prevention of bone complications was also conducted.

## Methods

### Study design and participants

This retrospective, longitudinal, cohort study used data from a German healthcare insurance claims database (Vilua Research Database, Vilua Healthcare GmbH), representative of the wider German population, recorded between 2007 and 2015. For that time period, data on patient demographics, primary and secondary care diagnoses (International Classification of Diseases [ICD]) and drug prescriptions (Anatomical Therapeutic Chemical Classification System [ATCCS]) were available for approximately 3.5 million patients (ICD codes used are shown in Supplementary Table 1). Detailed inpatient and outpatient procedure data were also available for a subset of approximately 2.5 million patients.

Patients eligible for inclusion were aged 18 years or older with a diagnosis of breast, prostate or lung cancer between 2007 and 2015, a bone metastasis diagnosis after 1 July 2011 and a first prescription for a bisphosphonate or denosumab after the initial bone metastasis diagnosis. For the exploratory analysis, patients were grouped according to whether they received treatment to prevent SREs ‘early’ or ‘late’ following a bone metastasis diagnosis. ‘Early initiation’ was defined as a first prescription in the 3 months after bone metastasis diagnosis, and ‘late initiation’ as a first prescription between 3 and 9 months after bone metastasis diagnosis. The two cohorts were adjusted for imbalances in baseline demographics with matched pairs, randomly selecting three patients from the early group for every patient in the late group (without replacement).

The study period was 1 July 2011–31 December 2015 and included a 6-month baseline period before the index date, defined as the date denosumab or bisphosphonate treatment was initiated. For the exploratory analysis cohort, the index date was defined as the diagnosis of bone metastases.

Follow-up data were collected on patients until December 2015, death or loss to follow-up. Patients with a diagnosis of hypercalcaemia before the bone metastasis diagnosis and individuals who had received bisphosphonates or denosumab in the 6-month period before the index date were excluded.

### Study outcomes

Primary outcomes included persistence, time to discontinuation, compliance, switch rates and therapy re-initiation usage patterns with denosumab or bisphosphonates at 6, 12 and 24 months following the index date. For this analysis, medication persistence was defined as a period of continuous treatment in which no gap between consecutive prescriptions exceeded 90 days. Medication compliance was defined as receiving 12 or more prescriptions per year. Switching was defined as receiving a prescription for an alternative agent in the 90 days after the last prescription of the initial therapy, and treatment re-initiation was defined as a repeat prescription for the initial agent after a gap of more than 90 days. All analyses were conducted for the initially prescribed therapy only. Exploratory outcomes included time to initiation of a bisphosphonate or denosumab across the three tumour types, and the association between time to therapy initiation and first (and subsequent) bone complication (defined using ICD codes for relevant diagnoses and/or inpatient procedures; Supplementary Table 1).

### Statistical analysis

All analyses performed in this study were descriptive. Categorical variables were summarised using the number, percentage and 95% confidence interval (CI), and continuous variables were summarised using the mean (± standard deviation) and median (95% CI). Kaplan–Meier graphs of persistence provided estimated median (95% CI) time to discontinuation of denosumab or bisphosphonate therapy; data were censored at the end of data collection, loss to follow-up or death, whichever occurred first. Data were not available for all therapies in all cancer types; subgroup analysis data (i.e. by tumour type and initiating treatment) were reported only for groups containing 30 patients or more. Sensitivity analyses were conducted using treatment gaps of 45, 60 and 120 days for persistence, switch rate and re-initiation patterns, and using 10, 11 and 13 prescriptions per year for compliance.

For the exploratory analyses, median (95% CI) time to medication initiation (denosumab and bisphosphonate therapy combined), stratified by tumour type, and time to first and subsequent bone complication, stratified by time to initiation category (early/late), were reported.

## Results

### Patient characteristics

In total, 82,070 patients with breast, prostate or lung cancer were screened in the database. Of these, 1130 were eligible for inclusion: 555 (49%) had breast cancer, 361 (32%) prostate cancer and 242 (21%) lung cancer (Supplementary Fig. 1). Baseline characteristics are presented in Table 1. Mean age was 65 years for patients with breast or lung cancer and 74 years for those with prostate cancer. At baseline, 25%, 17% and 20% of patients with breast, prostate and lung cancer, respectively, had experienced a previous bone complication. Renal disease was present in 8%, 23% and 16% of patients with breast, prostate and lung cancer, respectively (Supplementary Table 1 provides ICD-10 codes used to define comorbidities). The most commonly prescribed bone-protecting therapies were zoledronic acid and denosumab in patients with breast cancer (48% and 28%, respectively) and prostate cancer (58% and 34%, respectively), and zoledronic acid and pamidronate in patients with lung cancer (63% and 17%, respectively); a lower proportion of patients with lung cancer received denosumab than those with breast and prostate cancer (15% compared with 28% and 34%, respectively).

**Table 1:** Baseline characteristics and index therapy for patients with bone metastases, secondary to solid breast cancer, prostate cancer or lung cancer tumours, who were initiated on bisphosphonate or denosumab therapy after bone metastases diagnosis, stratified by solid tumour type and primary/exploratory analysis populations

Baseline characteristic	Primary analysis cohort				Exploratory analysis cohort			
	All^a^	Breast cancer	Prostate cancer	Lung cancer	All^a^	Breast cancer	Prostate cancer	Lung cancer
Patients, *N*	1130	555	361	242	2134	844	728	639
Female (%)	57.8	100.0	0.0	43.8	51.1	100.0	0.0	43.4
Age, mean (SD)	67.8 (11.4)	64.9 (12.8)	74.4 (7.9)	64.5 (10.0)	70.6 (11.6)	68.5 (13.1)	76.0 (8.4)	67.0 (10.3)
CCI, mean	9.9	9.44	10.32	10.19	–	–	–	–
Bone complication: any (%)	21.7	24.5	16.6	20.3	23.1	19.3	15.8	27.2
Pathologic fracture (%)	13.6	15.9	9.9	11.9	15.3	12.2	9.1	9.2
Radiotherapy (%)	7.2	7.9	5.8	7.0	6.7	7.4	5.6	18.6
Spinal cord compression (%)	2.4	1.4	1.6	2.9	2.9	1.8	2.6	2.7
Osteoporosis (%)	13.8	19.8	8.0	7.4	–	–	–	–
Renal disease (%)	14.3	7.8	22.9	16.1	–	–	–	–
CVD (%)	11.2	8.5	15.8	10.7	–	–	–	–
Cancer treatment (%)	45.8	41.9	62.1	33.1	–	–	–	–
Denosumab, *n* (%)	308 (27.3)	154 (27.7)	123 (34.1)	37 (15.3)	231 (10.8)	113 (13.4)	103 (14.2)	18 (2.8)
Zoledronic acid, *n* (%)	616 (54.5)	267 (48.1)	210 (58.2)	153 (63.2)	493 (23.1)	208 (24.6)	194 (26.7)	103 (16.1)
Ibandronate, *n* (%)	81 (7.2)	65 (11.7)	7 (1.9)	10 (4.1)	70 (3.3)	56 (6.6)	7 (1.0)	8 (1.3)
Pamidronate, *n* (%)	120 (10.6)	65 (11.7)	20 (0.6)	42 (17.4)	84 (3.9)	50 (5.9)	16 (2.2)	23 (3.6)
Clodronate, *n* (%)	3 (0.3)	3 (0.5)	0 (0.0)	0 (0.0)	7 (0.3)	2 (0.2)	3 (0.4)	3 (0.5)
None, *n* (%)	2 (0.2)	0 (0.0)	0 (0.0)	0 (0.0)	1249 (58.5)	415 (49.2)	405 (55.6)	484 (75.7)

*CCI* Charlson Comorbidity Index, *CVD* cardiovascular disease, *SD* standard deviation

^a^The total number of patients comprises the distinct number of patients with a diagnosis of prostate, breast or lung cancer. Patients with a diagnosis of more than one cancer type (e.g. prostate and lung cancer) were not counted twice in the total value

### Persistence

**Fig. 1:**
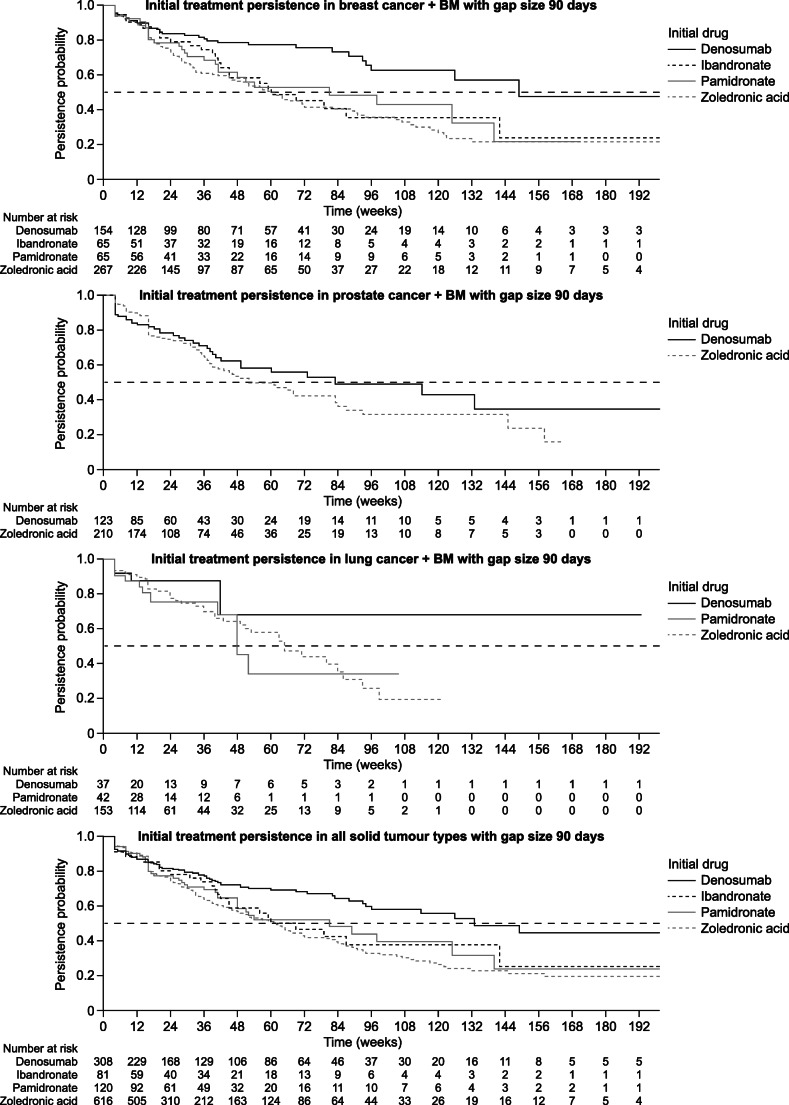
Kaplan–Meier analysis of medication persistence, stratified by index date therapy and solid tumour type. Medication persistence was analysed for the index date therapy only. Data shown here were calculated using a 90-day gap period. Patients were censored at the end of data collection, loss to follow-up or death. BM, bone metastases

Persistence was higher among patients receiving denosumab than among those receiving a bisphosphonate, across all tumour types and endpoints (Table 2). At 12 months, persistence among patients with breast cancer was 78%, 58%, 56% and 54% for denosumab, ibandronate, pamidronate and zoledronic acid, respectively. For patients with prostate cancer, 12-month persistence was 58% for denosumab and 50% for zoledronic acid. For those with lung cancer, 12-month persistence was 68%, 34% and 60% for denosumab, pamidronate and zoledronic acid, respectively. In Kaplan–Meier analyses, the probability of discontinuation was lower with denosumab than with any bisphosphonate, in all solid tumour types (Fig. 1). In patients with breast cancer, median time to discontinuation was 149 weeks for denosumab compared with 81 weeks for pamidronate and 60 weeks each for ibandronate and zoledronic acid. For patients with prostate cancer, median time to discontinuation was 83 weeks for denosumab compared with 52 weeks for zoledronic acid. For patients with lung cancer, median time to discontinuation was 48 weeks for pamidronate and 65 weeks for zoledronic acid; with denosumab, the median was not reached (NR; i.e. at no time point during follow-up had ≥ 50% of observable patients discontinued treatment).

**Table 2:** Time to discontinuation and medication persistence at 6, 12 and 24 months, stratified by solid tumour type and index date medication (treatment gap, 90 days)^a^

Gap size 90 days	Breast cancer	Prostate cancer	Lung cancer
Time to discontinuation, median (95% CI) weeks			
Denosumab	149 (126–NR)	83 (49–NR)	NR (42–NR)
Ibandronate	60 (45–NR)	–	–
Pamidronate	81 (41–NR)	–	48 (48–NR)
Zoledronic acid	60 (48–72)	52 (41–83)	65 (51–93)
6 months: % (95% CI) persistent			
Denosumab	83.7 (77.8–90.1)	76.8 (69.0–85.4)	87.5 (76.4–100.0)
Ibandronate	79.1 (69.2–90.5)	–	–
Pamidronate	78.4 (68.6–89.6)	–	75.4 (61.0–93.1)
Zoledronic acid	70.7 (65.0–76.9)	73.7 (67.7–80.3)	76.1 (68.4–84.8)
12 months: % (95% CI) persistent			
Denosumab	77.5 (70.4–85.3)	58.0 (47.5–70.9)	68.1 (46.8–99.0)
Ibandronate	58.3 (45.2–75.1)	–	–
Pamidronate	55.8 (43.3–72.0)	–	33.9 (14.5–79.6)
Zoledronic acid	53.6 (46.9–61.3)	49.7 (41.5–59.4)	60.1 (49.6–72.7)
24 months: % (95% CI) persistent			
Denosumab	62.8 (51.6–76.4)	49.2 (37.3–64.8)	68.1 (46.8–99.0)
Ibandronate	35.5 (21.8–57.9)	–	–
Pamidronate	43.0 (28.8–64.1)	–	33.9 (14.5–79.6)
Zoledronic acid	35.6 (28.3–44.8)	31.4 (22.2–44.5)	19.2 (8.2–44.7)

*CI* confidence interval, *NR* not reached

^a^Subgroup analysis data (i.e. by tumour type and initiating treatment) were reported only for those groups containing 30 or more patients. Therefore, data were not available for all therapies in all cancer types because some therapies were not prescribed or were infrequently prescribed in certain cancer types

### Compliance and switch rates

At 12 months, compliance was higher and switch rates were lower among patients initiated on denosumab than bisphosphonates in all tumour types (Table 3). The proportions of patients with breast cancer who had received 12 prescriptions in the year after the index date were 75%, 42%, 48% and 48% for denosumab, ibandronate, pamidronate and zoledronic acid, respectively. Compliance was 47% for denosumab and 36% for zoledronic acid in patients with prostate cancer, and 51% for zoledronic acid in those with lung cancer. Compliance data for patients with lung cancer who were initiated on denosumab are not presented, because this subgroup contained fewer than 30 patients. In patients with breast cancer, switch rates were 5%, 14%, 14% and 19% for denosumab, ibandronate, pamidronate and zoledronic acid, respectively. Switch rates were 2% for denosumab and 11% for zoledronic acid in patients with prostate cancer, and 3%, 12% and 7% for denosumab, pamidronate and zoledronic acid, respectively, in patients with lung cancer.

**Table 3:** 12-month medication compliance and switch and re-initiation rates, stratified by solid tumour type and index date medication (treatment gap 90 days)

	Breast cancer	Prostate cancer	Lung cancer
Compliance (12 prescriptions/year), % (95% CI)			
Denosumab	74.7 (63.6–83.8)	46.8 (32.1–61.9)	–
Ibandronate	42.4 (25.5–60.8)	–	–
Pamidronate	48.5 (30.8–66.5)	–	–
Zoledronic acid	48.2 (39.7–56.8)	36.1 (25.9–47.1)	51.2 (35.5–66.7)
Switch, % (95% CI)^a^			
Denosumab	4.6 (1.85–9.14)	2.4 (0.5–6.9)	2.7 (0.1–14.2)
Ibandronate	13.9 (6.5–24.7)	–	–
Pamidronate	13.9 (6.5–24.7)	–	11.9 (3.9–25.6)
Zoledronic acid	18.7 (14.2–23.9)	10.9 (7.1–15.9)	6.5 (3.2–11.7)
Re-initiation, % (95% CI)^b^			
Denosumab	10.4 (6.1–16.3)	15.5 (9.6–23.1)	10.8 (3.0–25.4)
Ibandronate	16.9 (8.8–28.3)	–	–
Pamidronate	10.8 (4.4–20.9)	–	2.4 (0.1–12.6)
Zoledronic acid	16.1 (11.9–21.1)	14.8 (10.3–20.3)	8.50 (4.6–14.1)

*CI* confidence interval

^a^A switch in therapy occurred when a prescription for an alternative agent was recorded in the 90 days after the last prescription of the initial therapy

^b^Treatment re-initiation was defined as a repeat prescription for the initial agent after a gap of more than 90 days

### Drug re-initiation rates

There were no clear class-specific patterns in drug re-initiation rates; however, rates were slightly higher among patients with breast cancer (10–17%) and prostate cancer (15%) than among those with lung cancer (2–11%) (Table 3).

### Sensitivity analysis

Sensitivity analysis using permissible gaps of 45, 60 and 120 days broadly confirmed the results of the 90-day analysis for persistence, switch rates and drug re-initiation. Similarly, analysis of compliance using 10, 11 and 13 prescriptions per year confirmed the results of the 12 prescriptions per year analysis (Supplementary Table 2).

### Time to therapy initiation (exploratory outcome)

**Fig. 2:**
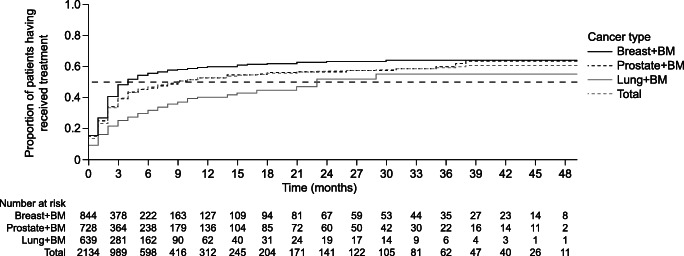
Time to initiation of denosumab or bisphosphonate therapy post bone metastases from solid tumour diagnosis, stratified by solid tumour type. Exploratory analysis of time to initiation of denosumab or bisphosphonate was analysed for all treatments combined. BM, bone metastases

Time to therapy initiation was recorded from the time of bone metastasis diagnosis to the time of a first prescription of denosumab or a bisphosphonate; data were combined for all treatment types. In total, 2211 patients were included in the time to initiation analysis. Baseline characteristics for these patients are presented in Table 1. In the Kaplan–Meier analysis, time to initiation of denosumab or a bisphosphonate was longer for patients with lung cancer, with a median (95% CI) delay between bone metastasis diagnosis and medication initiation of 23 (17–NR) months compared with 4 (3–5) months and 9 (7–14) months for patients with breast and prostate cancer, respectively (Fig. 2).

### Time to first and subsequent bone complication (exploratory outcome)

**Fig. 3:**
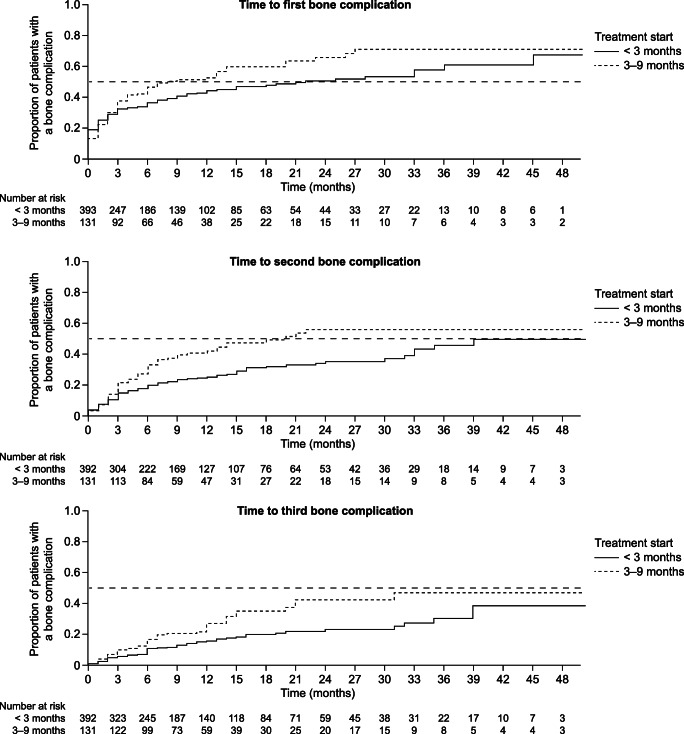
Time to first and subsequent bone complications in patients with solid tumours, stratified by time to denosumab/bisphosphonate initiation (early [≤ 3 months]/late [> 3–9 months]). Exploratory analysis of time to first or subsequent bone complication was analysed for all treatments combined

Patients who had received treatment to prevent bone complications within 9 months of bone metastasis diagnosis were eligible for the time to bone complication analysis (*n* = 971). To adjust for imbalances in baseline characteristics between patients receiving early (≤ 3 months) or late (> 3–9 months) treatment, the two cohorts were stratified by tumour type and presence of osteoporosis at baseline using a matched pair method without replacement. Following stratification, 592 patients were included in the analysis; 444 started the treatment early and 148 started late. Baseline characteristics were similar between patients initiating the treatment early and those initiating late (Supplementary Table 3): the proportions of patients who had experienced bone complications at baseline were 22% and 23%, respectively, and mean age was 70 years for both groups. The median (95% CI) time to first bone complication was 19 (12–33) months among early initiators and 7 (4–20) months among late initiators; median time to second bone complication was 39 (33–NR) months and 21 (13–NR) months, respectively. Median time to third bone complication was not reached for both groups (Fig. 3).

## Discussion

This study updates the limited evidence available on bisphosphonate and denosumab usage patterns in the current anti-tumour treatment landscape, by providing real-world data from patients with bone metastases from breast, prostate and lung cancer diagnosed between 2011 and 2015 in Germany. Across all tumour types and time points, persistence with denosumab was higher than with any bisphosphonate. Persistence and compliance with denosumab were lower for patients with prostate cancer than for those with breast cancer. This may reflect the male gender and older age of these patients, factors that have previously been shown to influence compliance [33]. In general, time to discontinuation was longer, compliance greater and switch rates lower in patients receiving denosumab than in those receiving bisphosphonates. The higher levels of persistence and compliance with denosumab than with bisphosphonates may be associated with improved outcomes in routine clinical practice [34]. Median time to discontinuation with denosumab was not reached and compliance data not reported in the lung cancer group owing to a small patient sample. These data may suggest a reluctance to prescribe supportive care treatments to patients who have a poor prognosis. Although there were no class-specific patterns for drug re-initiation, our data show that treatments were continued even after large gaps. The exploratory analyses suggest that prompt initiation of bone-protecting therapy after bone metastasis diagnosis may delay time to first and subsequent bone complications.

The denosumab and bisphosphonate usage patterns in our study are similar to those found in the two OSCER studies conducted in the USA in patients with bone metastases from breast, prostate and lung cancer [33, 34]. In a 1-year study of 3569 patients, those prescribed denosumab received a higher median number of annual doses and were less likely to switch to an alternative therapy than those initially prescribed a bisphosphonate [33]. These results were confirmed in a 3-year study in which patients receiving denosumab were less likely to switch agents and more likely to demonstrate compliance with treatment at 1, 2 and 3 years across all solid tumour subtypes. Median time to non-persistence was also significantly longer for denosumab than for zoledronic acid [34].

Although the determinants of persistence and compliance can only be inferred, there are several potential reasons why persistence and compliance are greater with denosumab in this study. First, denosumab has demonstrated superiority to zoledronic acid in preventing bone complications in patients with solid tumours; therefore, physicians may be more motivated to ensure compliance [23]. Second, in an integrated analysis of three phase 3 clinical trials in patients with bone metastases from solid tumours, denosumab was more effective than zoledronic acid at extending the time to significant increases in pain and use of strong opioids [29]. Third, discrete-choice studies have suggested that patients and physicians prefer the 4-weekly SC injection of denosumab to the 3–4-weekly IV infusion of bisphosphonates [35, 36]. Finally, side effects, particularly renal toxicity and acute-phase reactions, are important factors in determining physician and patient treatment preferences [35, 36]. The use of IV bisphosphonates requires routine renal monitoring and dose adjustments, complicating the care of these patients [14, 37]. In contrast, denosumab is not excreted by the kidneys and does not require renal monitoring, which may make optimum compliance and persistence easier to achieve [15]. Given that this study comprised an elderly patient population (mean age ≥ 65 years), poorer persistence and compliance recorded for patients receiving IV bisphosphonates may reflect physicians’ decisions to skip or delay doses because of renal impairment [35, 36]. Of note, 3-monthly zoledronic acid infusions have been shown to be non-inferior to monthly infusions in clinical trials of patients with multiple myeloma and breast or prostate cancer with bone metastasis [38, 39]. It is possible that less frequent dosing may affect persistence and compliance with zoledronic acid; however, this has not been formally assessed in clinical studies, and this dosing regimen is currently off-label.

Exploratory analysis on the time to denosumab or bisphosphonate initiation showed large differences among tumour types, with a much longer median time to initiation recorded for patients with lung cancer than for those with breast or prostate cancer. The shortest time to treatment initiation was seen in those with breast cancer (median time 4 months). Similarly, a real-world study assessing the treatment of bone metastases in patients with breast cancer across six European countries, including Germany, showed that 81% of patients treated with denosumab or bisphosphonates received therapy in the 3 months after bone metastasis diagnosis [40]. The long median time to treatment initiation in patients with lung cancer (23 months) may reflect poor prognosis in this patient group; studies of other cancer types have shown that physicians cite poor prognosis as a reason for not initiating treatment to prevent bone complications [40]. Our data suggest that improvements in bone healthcare are needed to ensure that all patients receive treatment at the time of bone metastasis diagnosis to prevent bone complications, in line with the current guidelines [5].

The main strength of this study was that it incorporated a large patient sample representative of the wider German population and, unlike the OSCER studies, was not limited to patients treated in the oncology clinic setting only, so improving the generalisability of the results. The study has some limitations. The study was descriptive and not designed to demonstrate significant differences in outcomes between different tumour types, drug classes or time to initiation subgroups; therefore, these results should be interpreted with some caution. As with all claims studies, data are based on prescription claims records and may not correspond to actual drug consumption. Additionally, determinants of differential usage patterns between agents can only be inferred. Furthermore, usage definitions must be meaningful; the 90-day gap definition for persistence and the 12 prescription definition for compliance were considered appropriate for this study and are in line with previously published studies [33, 34]. Sensitivity analyses using alternative gaps (45, 60 and 120 days, and 10, 11 and 13 prescriptions per year) broadly confirmed the result of the primary analysis. The time between bone metastasis diagnosis and treatment initiation may be underestimated; the occurrence of a bone complication, or initiation of denosumab or bisphosphonate therapy, may trigger the (retrospective) recording of bone metastases, resulting in a delay between the documentation of the diagnosis in the patient’s medical chart and its appearance in their administrative claims record. Finally, there are inherent difficulties in defining bone complications using claims data, and the exploratory analyses on the time to first bone complication should be interpreted with caution.

## Conclusions

In this real-world study of German clinical practice, patients initiated on denosumab following a bone metastasis diagnosis from breast, prostate or lung cancer had higher medication persistence, longer time to discontinuation, improved compliance and lower switch rates compared with those initiated on a bisphosphonate. Median time to first and subsequent bone complications was shorter for late vs early initiators of either agent, thereby supporting clinical recommendations to introduce bone-protecting therapy at the time of bone metastasis diagnosis to provide optimal patient care and maximise patient QoL.

## Electronic supplementary material

Supplementary Fig. 1 Flow diagram of patient enrolment. *BC* breast cancer, *LC* lung cancer, *PC* prostate cancer. The total number of patients comprises the distinct number of patients with a diagnosis of prostate, breast or lung cancer. Patients with a diagnosis of more than one cancer type (e.g. prostate and lung cancer) were not counted twice in the total value (PNG 65 kb)

High Resolution Image (EPS 2106 kb)

Supplementary Table 1 International Classification of Diseases and German Procedure Classification codes for primary study definitions. *ICD-10* International Classification of Diseases, 10th revision, *OPS* Operationen- und Prozedurenschlüssel (DOCX 13 kb)

Supplementary Table 2 Sensitivity analysis using different lengths of treatment gaps (discontinuation, persistence, switch, re-initiation) or different numbers of prescriptions received per year (compliance). *CI* confidence interval, *NR* not reached (DOCX 16 kb)

Supplementary Table 3 Baseline characteristics of the unmatched and matched populations included in the exploratory time to first and subsequent bone complication analysis stratified by early/late initiation of treatment. *CCI* Charlson Comorbidity Index, *SD* standard deviation, *SRE* skeletal-related event. ^a^Only includes patients who also had a diagnosis of bone metastases (DOCX 13 kb)

## Data Availability

The data sets generated and analysed during the current study are not publicly available, but they are available from the corresponding author, who has full control of all primary data, on reasonable request.
