# Supplementary figures and images for: Real-world use of denosumab and bisphosphonates in patients with solid tumours and bone metastases in Germany
Source: Support Care Cancer. 2020 Feb 21;28(11):5223–33. doi: 10.1007/s00520-020-05357-5 (PMC7547046; doi:10.1007/s00520-020-05357-5)

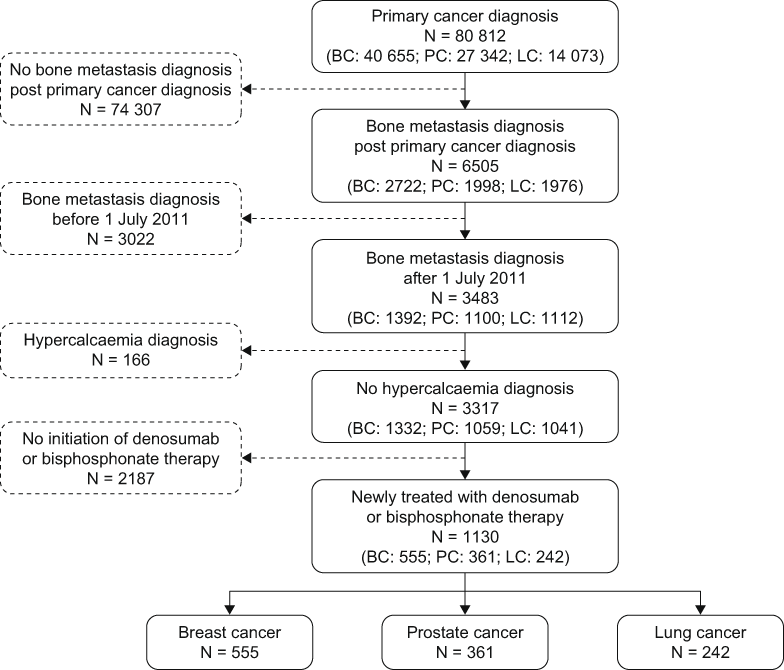

Supplement: Supplementary file 1 — Flow diagram of patient enrolment. BC breast cancer, LC lung cancer, PC prostate cancer. The total number of patients comprises the distinct number of patients with a diagnosis of prostate, breast or lung cancer. Patients with a diagnosis of more than one cancer type (e.g. prostate and lung cancer) were not counted twice in the total value (PNG 65 kb) [file 520_2020_5357_Fig4_ESM.png]
