# Supplementary material for: Real-world use of denosumab and bisphosphonates in patients with solid tumours and bone metastases in Germany
Source: Support Care Cancer. 2020 Feb 21;28(11):5223–33. doi: 10.1007/s00520-020-05357-5 (PMC7547046; doi:10.1007/s00520-020-05357-5)
Supplement: Supplementary file 3 — International Classification of Diseases and German Procedure Classification codes for primary study definitions. ICD-10 International Classification of Diseases, 10th revision, OPS Operationen- und Prozedurenschlüssel (DOCX 13 kb) [file 520_2020_5357_MOESM2_ESM.docx]

| Condition | Code type | ICD/medication code |
| --- | --- | --- |
| Cancer type |  |  |
| Breast cancer | ICD-10 | C50 |
| Prostate cancer | ICD-10 | C61 |
| Lung cancer | ICD-10 | C34 |
| Solid tumour and newly diagnosed bone metastases | ICD-10 | C79.5 |
| Skeletal-related event type |  |  |
| Pathologic fracture | ICD-10 | M48.5; M84.4; M90.7; S12; S22; S32; S42; S52; S62; S72; S82; S92 |
| Spinal cord compression | ICD-10 | G95.2; G95.8; G95.9; M43.9; M47.12; M47.14; M47.15; M47.16; M47.19; M48.5; M50.0; M50.9; M51.0; M54 |
| Surgery to bone | OPS | 5-79; 5-790; 5-791; 5-792; 5-81; 5-836 |
| Radiation to bone | ICD-10 | Z51.0 |
|  | OPS | 5-921.6; 8-52; 8-520; 8-521; 8-522; 8-523; 8-524; 8-525; 8-526; 8-527; 8-528; 8-529; 8-52a; 8-52b; 8-52c; 8-52d; 8-530.1 |
| Comorbidities |  |  |
| Renal disease | ICD-10 | I12.0; I13.1; N03.2-N03.7; N05.2-N05.7; N18.x; N19.x; N25.0; Z49.0-Z49.2; Z94.0; Z99.2 |
| Cardiovascular disease |  |  |
| Myocardial infarction | ICD-10 | I21.x; I22.x; I25.2 |
| Congestive heart failure | ICD-10 | I09.9; I11.0; I13.0; I13.2; I25.5; I42.0; I42.5-I42.9; I43.x; I50.x; P29.0 |
| Peripheral vascular disease | ICD-10 | I70.x; I71.x; I73.1; I73.8; I73.9; I77.1; I79.0; I79.2; K55.1; K55.8; K55.9; Z95.8; Z95.9 |
| Cerebrovascular disease | ICD-10 | G45.x; G46.x; H34.0; I60.x-I69.x |
