# Supplementary material for: Real-world use of denosumab and bisphosphonates in patients with solid tumours and bone metastases in Germany
Source: Support Care Cancer. 2020 Feb 21;28(11):5223–33. doi: 10.1007/s00520-020-05357-5 (PMC7547046; doi:10.1007/s00520-020-05357-5)
Supplement: Supplementary file 4 — Sensitivity analysis using different lengths of treatment gaps (discontinuation, persistence, switch, re-initiation) or different numbers of prescriptions received per year (compliance). CI confidence interval, NR not reached (DOCX 16 kb) [file 520_2020_5357_MOESM3_ESM.docx]

|  | Length of treatment gap/number of prescriptions per year | Breast cancer | Prostate cancer | Lung cancer |  |
| --- | --- | --- | --- | --- | --- |
| Time to discontinuation, median (95% CI) weeks | | | | | |
| Denosumab | 45 days | 23 (21–38) | 22 (13–35) | 14 (10–NR) |  |
|  | 60 days | 92 (53–NR) | 39 (29–83) | 56 (33–NR) |  |
|  | 120 days | NR (149–NR) | NR (83–NR) | NR (NR–NR) |  |
| Ibandronate | 45 days | 20 (25–53) | – | – |  |
|  | 60 days | 45 (41–87) | – | – |  |
|  | 120 days | 142 (56–NR) | – | – |  |
| Pamidronate | 45 days | 20 (16–40) | – | 16 (11–NR) |  |
|  | 60 days | 40 (28–61) | – | 41 (16–NR) |  |
|  | 120 days | 125 (54–NR) | – | NR (52–NR) |  |
| Zoledronic acid | 45 days | 28 (23–30) | 24 (16–30) | 19 (16–24) |  |
|  | 60 days | 32 (29–46) | 33 (28–36) | 33 (24–51) |  |
|  | 120 days | 72 (64–110) | 84 (76–NR) | 80 (65–NR) |  |
| 12-month persistence, % (95% CI) | | | | | |
| Denosumab | 45 days | 32.9 (25.2–43.0) | 24.9 (16.1–38.5) | 13.1 (2.6–67.0) |  |
|  | 60 days | 59.3 (51.0–68.9) | 43.7 (33.6–56.8) | 56.6 (37.2–86.3) |  |
|  | 120 days | 82.9 (76.4–89.9) | 70.7 (60.9–82.2) | 81.1 (62.0–100.0) |  |
| Ibandronate | 45 days | 34.0 (22.2–52.3) | – | – |  |
|  | 60 days | 49.6 (36.8–66.9) | – | – |  |
|  | 120 days | 62.8 (50.0–78.9) | – | – |  |
| Pamidronate | 45 days | 21.3 (12.3–36.8) | – | 11.6 (2.3–59.2) |  |
|  | 60 days | 39.5 (27.8–56.1) | – | 9.0 (1.5–55.3) |  |
|  | 120 days | 64.1 (51.0–80.5) | – | 50.9 (26.9–96.3) |  |
| Zoledronic acid | 45 days | 28.2 (31.9–45.9) | 17.7 (12.0–26.2) | 8.3 (3.6–19.3) |  |
|  | 60 days | 38.2 (31.9–45.9) | 27.2 (20.3–36.6) | 34.4 (25.1–47.2) |  |
|  | 120 days | 62.9 (56.2­–70.3) | 63.6 (55.9–72.4) | 73.3 (63.4–84.8) |  |
| 12-month switch rate, % (95% CI) | | | | | |
| Denosumab | 45 days | 3.3 (1.1–7.4) | 1.6 (0.2–5.8) | 0 (0–9.5) |  |
|  | 60 days | 4.6 (1.9–9.1) | 1.6 (0.2–5.8) | 0 (0–9.5) |  |
|  | 120 days | 4.6 (1.9–9.1) | 4.1 (1.3–9.2) | 2.7 (0.1–14.2) |  |
| Ibandronate | 45 days | 6.2 (1.7–15.0) | – | – |  |
|  | 60 days | 10.8 (4.4–20.9) | – | – |  |
|  | 120 days | 15.4 (7.6–26.5) | – | – |  |
| Pamidronate | 45 days | 10.8 (4.4–20.9) | – | 7.1 (1.5–19.5) |  |
|  | 60 days | 12.3 (5.5–22.8) | – | 9.5 (2.7–22.6) |  |
|  | 120 days | 13.9 (6.5–24.7) | – | 11.9 (3.9–25.6) |  |
| Zoledronic acid | 45 days | 12.7 (8.9–17.3) | 6.7 (3.7–10.9) | 3.3 (1.1–7.5) |  |
|  | 60 days | 14.2 (10.3–19.0) | 9.1 (5.5–13.8) | 5.9 (2.7–10.9) |  |
|  | 120 days | 19.5 (14.9–24.7) | 11.4 (7.5–16.5) | 6.5 (3.2–11.7) |  |
| 12-month re-initiation rate, % (95% CI) | | | | | |
| Denosumab | 45 days | 45.5 (37.4–53.7) | 41.5 (32.7–50.7) | 37.8 (22.5–55.2) |  |
|  | 60 days | 23.4 (16.9–30.9) | 27.6 (19.9–36.4) | 21.6 (9.8–38.2) |  |
|  | 120 days | 5.2 (2.3–9.9) | 8.1 (3.9–14.4) | 0 (0–9.49) |  |
| Ibandronate | 45 days | 41.5 (29.4–54.4) | – | – |  |
|  | 60 days | 27.7 (17.3–40.2) | – | – |  |
|  | 120 days | 9.2 (3.5–19.0) | – | – |  |
| Pamidronate | 45 days | 44.6 (32.3–57.5) | – | 26.2 (13.9–42.0) |  |
|  | 60 days | 30.8 (19.9–43.5) | – | 11.9 (3.9–25.6) |  |
|  | 120 days | 7.7 (2.5–17.1) | – | 2.4 (0.1–12.6) |  |
| Zoledronic acid | 45 days | 42.3 (36.3–48.5) | 40.5 (33.8–47.5) | 35.3 (27.8–43.4) |  |
|  | 60 days | 32.6 (27.0–38.6) | 31.9 (25.7–38.7) | 23.5 (17.1–31.1) |  |
|  | 120 days | 9.7 (6.5–13.9) | 7.1 (4.1–11.5) | 5.9 (2.7–10.9) |  |
| 12-month compliance, % (95% CI) | | | | | |
| Denosumab | 10 prescriptions/year | 86.1 (76.5–92.8) | 68.1 (52.9–80.9) | – |  |
|  | 11 prescriptions/year | 82.3 (72.1–89.9) | 61.7 (46.4–75.5) | – |  |
|  | 13 prescriptions/year | 45.6 (34.3–57.2) | 21.3 (10.7–35.7) | – |  |
| Ibandronate | 10 prescriptions/year | 72.7 (54.5–86.7) | – | – |  |
|  | 11 prescriptions/year | 60.6 (42.1–77.1) | – | – |  |
|  | 13 prescriptions/year | 30.3 (15.6–48.7) | – | – |  |
| Pamidronate | 10 prescriptions/year | 66.7 (48.2–82.0) | – | – |  |
|  | 11 prescriptions/year | 63.6 (45.1–79.6) | – | – |  |
|  | 13 prescriptions/year | 33.3 (17.9–51.8) | – | – |  |
| Zoledronic acid | 10 prescriptions/year | 59.7 (51.1–67.9) | 58.1 (47.0–68.7) | 67.4 (51.5–80.9) |  |
|  | 11 prescriptions/year | 54.7 (46.0–63.1) | 47.7 (36.8–58.7) | 58.1 (42.1–72.9) |  |
|  | 13 prescriptions/year | 30.2 (22.7–38.6) | 23.3 (14.8–33.6) | 30.2 (17.2–46.1) |  |
