# Supplementary material for: Real-world use of denosumab and bisphosphonates in patients with solid tumours and bone metastases in Germany
Source: Support Care Cancer. 2020 Feb 21;28(11):5223–33. doi: 10.1007/s00520-020-05357-5 (PMC7547046; doi:10.1007/s00520-020-05357-5)
Supplement: Supplementary file 5 — Baseline characteristics of the unmatched and matched populations included in the exploratory time to first and subsequent bone complication analysis stratified by early/late initiation of treatment. CCI Charlson Comorbidity Index, SD standard deviation, SRE skeletal-related event. aOnly includes patients who also had a diagnosis of bone metastases (DOCX 13 kb) [file 520_2020_5357_MOESM4_ESM.docx]

|  | Unmatched population | | Matched population | |
| --- | --- | --- | --- | --- |
| Characteristic | Early (≤3 months) *N* = 823 | Late (>3–9 months) *N* = 148 | Early (≤3 months) *N* = 444 | Late (>3–9 months) *N* = 148 |
| Female, *n* (%) | 452 (54.9) | 70 (47.3) | 211 (47.5) | 70 (47.3) |
| Mean age, years (SD) | 69.7 (11.4) | 70.1 (10.9) | 70.4 (11.1) | 70.1 (10.9) |
| Mean CCI (SD) | 10.1 (2.1) | 10.2 (2.1) | 10.2 (2.2) | 10.2 (2.1) |
| Cancer type, *n* (%)^a^ |  |  |  |  |
| Breast cancer | 370 (45.0) | 52 (35.1) | 156 (35.1) | 52 (35.1) |
| Prostate cancer | 262 (31.8) | 52 (35.1) | 156 (35.1) | 52 (35.1) |
| Lung cancer | 134 (16.2) | 27 (18.2) | 81 (18.2) | 27 (18.2) |
| Other | 57 (6.9) | 17 (11.5) | 51 (11.5) | 17 (11.5) |
| SRE, *n* (%) | 173 (21.0) | 34 (23.0) | 97 (21.8) | 34 (23.0) |
| Osteoporosis, *n* (%) | 138 (16.8) | 17 (11.5) | 51 (11.5) | 17 (11.5) |
| Renal disease, *n* (%) | 147 (17.9) | 32 (21.6) | 94 (21.2) | 32 (21.6) |
| Cardiovascular disease, *n* (%) | 101 (12.3) | 20 (13.5) | 59 (13.3) | 20 (13.5) |
